# Site-specific Relaxase Activity of a VirD2-like Protein Encoded within the *tfs4* Genomic Island of Helicobacter pylori*[Fn FN2]

**DOI:** 10.1074/jbc.M113.496430

**Published:** 2013-07-29

**Authors:** Jane I. Grove, Maher N. Alandiyjany, Robin M. Delahay

**Affiliations:** From the Centre for Biomolecular Sciences and Nottingham Digestive Diseases Centre, National Institute for Health Research Biomedical Research Unit, University of Nottingham, Nottingham NG7 2RD, United Kingdom

**Keywords:** Bacterial Conjugation, Bacterial Pathogenesis, DNA Enzymes, Helicobacter pylori, Protein-DNA Interaction, Protein Domains, Integrating Conjugative Element, Relaxase, Tfs4, VirD2

## Abstract

Four different type IV secretion systems are variously represented in the genomes of different *Helicobacter pylori* strains. Two of these, encoded by *tfs3* and *tfs4* gene clusters are contained within self-transmissible genomic islands. Although chromosomal excision of *tfs4* circular intermediates is reported to be dependent upon the function of a *tfs4*-encoded XerD tyrosine-like recombinase, other factors required for transfer to a recipient cell have not been demonstrated. Here, we characterize the functional activity of a putative *tfs4*-encoded VirD2-like relaxase protein. Tfs4 VirD2 was purified as a fusion to maltose-binding protein and demonstrated to bind and nick both supercoiled duplex DNA and oligonucleotides *in vitro* in a manner dependent upon the presence of Mg^2+^ but independently of any auxiliary proteins. Unusually, concentration-dependent nicking of duplex DNA appeared to require only transient protein-DNA interaction. Although phylogenetically distinct from established relaxase families, site-specific cleavage of oligonucleotides by Tfs4 VirD2 required the nick region sequence 5′-ATCCTG-3′ common to transfer origins (*oriT*) recognized by MOB_P_ conjugative relaxases. Cleavage resulted in covalent attachment of MBP-VirD2 to the 5′-cleaved end, consistent with conventional relaxase activity. Identification of an *oriT*-like sequence upstream of *tfs4 virD2* and demonstration of VirD2 protein-protein interaction with a putative VirC1 relaxosome component indicate that transfer initiation of the *tfs4* genomic island is analogous to mechanisms underlying mobilization of other integrated mobile elements, such as integrating conjugative elements, requiring site-specific targeting of relaxase activity to a cognate *oriT* sequence.

## Teaser

**Background:** The complement of factors involved in mobilization of the *Helicobacter pylori* disease-associated *tfs4* genomic island are presently unknown.

**Results:** *tfs4* encodes a VirD2-like relaxase with distinctive DNA binding and nicking activity.

**Conclusion:** Tfs4 VirD2 probably initiates mobilization of *tfs4* by specific interaction at a chromosomal transfer origin sequence.

**Significance:** Tfs4 VirD2-mediated mobilization of *tfs4* may increase pathogenic potential of *H. pylori* strains.

## Introduction

*Helicobacter pylori* is typically acquired in childhood and persistently colonizes the gastric mucosa of approximately half of the human population. It has the potential to cause a range of gastroduodenal diseases, including gastritis, peptic ulcer disease, mucosa-associated lymphoid tissue lymphoma, and gastric carcinoma (1–3). However, although infection is widespread and persistent, a complex interplay between multiple host, bacterial, and environmental factors determines that only about 20% of infected individuals will develop severe disease (3). A particular characteristic of *H. pylori* considered to contribute to its longevity in the host is its exceptional genetic variability, thought to be primarily a consequence of mutation and frequent intra- and intergenomic recombination events (4–6). With respect to the latter, the precise mechanisms of gene acquisition by horizontal transfer are not well defined but are considered to comprise both transformation and conjugative processes (7, 8). As a consequence of these collective mechanisms, an estimated 2–9% of the genome from any given isolate may be strain-specific, contributing to a predicted pan-genome approximately 4 times larger than the core genome (9).

Many strain-specific genes are localized in regions of genome diversity termed “plasticity zones” (PZs),^2^ which vary in number in the *H. pylori* chromosome and characteristically display low G + C content (10–12). Differences in PZ carriage and gene content may endow different *H. pylori* isolates with a selective advantage for niche colonization and increased virulence potential. Indeed, several genetic markers encoded specifically within PZs have been reported to associate with an increased risk for particular gastroduodenal diseases. These include homologues of strain J99 genes *jhp0947*, *jhp0940*, *jhp0945*, and *jhp0917/918* (13–16). The latter is known to comprise a single reading frame in most isolates where it occurs and, through its positive association with the incidence of duodenal ulcer in several geographically distinct patient populations, has been termed “duodenal ulcer-promoting” gene (*dupA*) (16). *dupA* has also been reported to increase survival at low pH and increase the production of IL-8 from gastric epithelial cells and IL-12 from monocytes (16). Although DupA function is unknown, it probably encodes a VirB4 ATPase (16) presumably associated with the activity of a type IV secretion system. Support for this notion is provided by analysis of recently completed genome sequences in which *dupA* is located proximal to a complement of other *vir*-homologous T4SS genes.

In certain strains of *H. pylori*, four distinct clusters of T4SS genes have been identified (11, 12). The *comB* cluster, common to all *H. pylori* strains, encodes a minimal complement of T4SS components specialized for DNA uptake during transformation (7) and more recently has also been implicated in the transfer of plasmids between *H. pylori* strains (8). The *cag* pathogenicity island encoding a second T4SS is an important virulence factor, mediating translocation of the host-stimulatory CagA effector and peptidoglycan fragments to the gastric epithelium (17–19). The last two clusters, termed *tfs3* and *tfs4* are contained within mobilizable elements described as either transferable genomic islands or conjugative transposons (10–12). The *tfs3* clusters in certain strain backgrounds have been reported to increase colonization fitness or up-regulate proinflammatory signaling from cultured epithelial cells, but an overarching phenotype remains elusive (11). The *tfs4* cluster has a complement of genes similar to that of *tfs3* and includes the disease marker *dupA*.

Recent work has demonstrated that large fragments of the *tfs4* island can be horizontally transferred in a manner dependent upon the activity of a XerD family tyrosine recombinase also encoded within the *tfs4* cluster (12). XerD excises the *tfs4* element at conserved flanking 5′-AAAGAATG-3′ motifs to generate a circular transfer intermediate that may subsequently be transferred to a recipient cell via the *tfs4*-encoded Tfs4 T4SS (12). Intermediate transfer steps are unknown; however, by analogy to conjugative mechanisms employed by both plasmids and other mobilizable genetic elements, such as integrating conjugative elements (ICEs), transfer probably also involves specific activity of an associated relaxase at a *cis*-acting origin of transfer (*oriT*) sequence comprising a *nic* cleavage site. Plasmid-encoded conjugative relaxases catalyze site- and strand-specific cleavage at *nic*, resulting in covalent attachment of the relaxase to the 5′-end of the nicked strand via a phosphotyrosyl linkage (20–23). Relaxases of both conjugal transposons and ICEs demonstrate similar activity, although few have been characterized to date (24–27). Targeting of specific relaxase activity to a cognate *oriT* sequence invariably requires the contribution of a varying number of auxiliary relaxosome proteins, which bind at *oriT* and facilitate *oriT* recognition and DNA processing by the relaxase (21, 28, 29). The relaxosome proteins are also integral to recruitment of the DNA-bound relaxase to a coupling protein for subsequent transfer via the membrane-embedded transfer machinery (30–32).

In addition to XerD and the T4SS structural *vir* gene complement, the *tfs4* element also encodes a putative VirD2-like relaxase, which we considered might function to initiate transfer of XerD-excised *tfs4* intermediates. To address this possibility, we studied the biochemical properties of Tfs4 VirD2, demonstrating it to have a distinctive *in vitro* site- and strand-specific nicking activity consistent with conjugative relaxase function. We additionally identified a putative *tfs4 oriT* region within *tfs4* and demonstrate interaction of Tfs4 VirD2 with a putative VirC1-like relaxosome protein. These studies suggest that the *tfs4* PZ cluster encodes a complete complement of proteins enabling self-transmission via a conjugative mechanism analogous to other self-transmissible mobile genetic elements.

## EXPERIMENTAL PROCEDURES

### 

#### 

##### Bacterial and Yeast Strains

*H. pylori* strain AB21 (33) was minimally passaged on blood agar plates (Oxoid) in a microaerobic environment. *Escherichia coli* strains XLI-Blue (*recA1 endA1 gyrA96 thi-1 hsdR17 supE44 relA1 lac* [F′ *proAB lacl*q*Z*Δ*M15* Tn*10* (TetR)]c), BL21(DE3)pLysS (F-*ompT hsdS*B (rB-mB−) *gal dcm* (DE3) pLysS (CmR)), and Shuffle (F′ *lac*, *pro*, *lacIQ*/Δ*(ara-leu)7697 araD139 fhuA2 lacZ*::*T7 gene1* Δ*(phoA)PvuII phoR ahpC galE (or U) galK* λ*att*::pNEB3-r1-*cDsbC* (Spec^R^, *lacI^q^*) Δ*trxB rpsL150*(Str^R^) Δ*gor* Δ*(malF)3*) were grown at 37 °C in Luria agar or broth supplemented with kanamycin (50 μg ml^−1^), ampicillin (50 μg ml^−1^), or chloramphenicol (30 μg ml^−1^) as required. *Saccharomyces cerevisiae* strain PJ69-4A (*MAT*α *trp1-901 leu2-3112 ura3-52 his3-200 gal4*Δ *gal80*Δ *LYS2*::*GAL1-HIS3 GAL2-ADE2 met2*::*GAL7-lacZ*) was grown at 30 °C and maintained in complete synthetic complete medium supplemented with 2% glucose (w/v).

##### Sequence and Phylogenetic Analyses

Genome sequences were retrieved from the NCBI database from where PSI-BLAST searches were also performed. Sequence comparison employed the EMBOSS Needle alignment tool, and identification of palindromic sequence used the EMBOSS Palindrome program. Coiled coil predictions were performed using COILS and Paircoil2. For phylogenetic analyses, 33 relaxase sequences, comprising 2–4 sequences representative of each of the different MOB clades (34), were downloaded from the NCBI protein database. A FASTA-formatted sequence file comprising the first 300 amino acids of each sequence (N-terminal relaxase domain) was aligned using the MEGA 4 implementation of ClustalW. Phylogenetic trees were calculated by MEGA 4 (35) using the neighbor-joining method (36). Bootstrap analysis was performed with 2000 resampled data sets from evolutionary distance, based on amino acid sequence alignments.

##### Cloning

Standard techniques for DNA manipulations were used in *E. coli* strain XL1-Blue. Genomic DNA was prepared from *H. pylori* strain AB21 after growth for 48 h on plates using a genomic DNA preparation kit (Sigma). Phusion polymerase (New England Biolabs) was used to amplify *H. pylori* DNA sequences according to the manufacturer's recommendations using primers listed in Table 1. The *virD2* gene was amplified with primers virD2F1 and virD2R1 for the full-length gene or virD2R2 for the relaxase domain only (Table 1) and then cloned directly into pGEM-TEasy or digested with BamHI and cloned into pMal-c2X (NEB) for expression with an N-terminal MBP fusion. The *virC1* gene was amplified with primers virC1F and virC1R and cloned into pET28a (Novagen). A *tfs4* fragment containing *virD2* and upstream intergenic region was amplified with primers virD2R1 and virD4F and cloned into pGEM-TEasy. For the yeast two-hybrid assay, *tfs4 virD2, virC1*, *0449*, and *0450* homologous genes were amplified from gDNA prepared from strain AB21 with the primers listed in Table 1. After digestion with BamHI for VirD2 and EcoRI plus BamHI for the other genes, the resulting fragments were then cloned into pGAD424 and pGBT9. All constructs were verified by sequencing, and *tfs4* gene sequences were deposited in GenBank^TM^ with accession numbers KF438085 (*virD2* region), KF438086 (*0450*), KF438087 (*0449*) and KF438088 (*virC1*).

**TABLE 1:** **Plasmids**

Plasmids	Relevant genotype and/or description	Reference or source
pET28a	T7 expression vector, Km^R^	Novagen
pMal-c2X	*malE* fusion vector, Ap^R^	New England Biolabs
pGEM-TEasy	High copy number cloning vector, Ap^R^	Promega
pSB14	RP4 *oriT*, Km^R^	Ref. 45
pGAD424	*ori*ColE1 *ori*2μ LEU1 P_ADH_::GAL4′ activator domain::MCS	Ref. 38
pGBT9	*ori*ColE1 *ori*2μ TRP1 P_ADH_::GAL4′ binding domain::MCS	Ref. 38
pRD200	pGEM-TEasy::*virD2 tfs4* from strain AB21	This study
pRD205	pGEMTEasy::*virD2 tfs4* + upstream intergenic flanking sequence from strain AB21	This study
pMal-D2	pMal::*virD2 tfs4* (1–1911 bp)	This study
pMal-D2(N)	pMal::*virD2 tfs4* (1–771 bp)	This study
pET28a-C1	pET28a::*virC1 tfs4* (1–656 bp)	This study
pGAD424-D2	pGAD424::*virD2 tfs4* (1–1911 bp)	This study
pGAD424-C1	pGAD424::*virC1 tfs4* (1–657 bp)	This study
pGAD424-0449	pGAD424::*0449 tfs4* (1–285 bp)	This study
pGAD424-0450	pGAD424::*0450 tfs4* (88–1098 bp)	This study
pGBT9-D2	pGBT9::*virD2 tfs4* (1–1911 bp)	This study
pGBT9-C1	pGBT9::*virC1 tfs4* (1–657 bp)	This study
pGBT9-0449	pGBT9::*0449 tfs4* (1–285 bp)	This study
pGBT9-0450	pGBT9::*0450 tfs4* (88–1098 bp)	This study

##### Protein Purification

MBP fusions were expressed in 500-ml cultures of *E. coli* Shuffle (New England Biolabs) in 2xYT medium (8 g of Bacto tryptone, 5 g of yeast extract, and 5 g of NaCl per 500 ml) and induced with 1 mm isopropyl β-d-1-thiogalactopyranoside in the presence of 0.2% glucose for 4 h at 25 °C. Bacteria were harvested and lysed by sonication in buffer A (50 mm Tris, 200 mm NaCl, 1 mm EDTA, 1 mm DTT, pH 7.5) in five 10-s bursts at an amplitude of 10 μm using a Soniprep 150 sonicator fitted with a 9.5-mm probe (MSE).

The soluble proteins were incubated with 1 ml of amylose resin (New England Biolabs) for 1 h at 4 °C, and then the column was washed with 30 ml of buffer A. Proteins were eluted in 4 ml of buffer A containing 10 mm maltose, 0.45 μm-filtered and diluted to 20 mm NaCl in TED buffer (50 mm Tris, pH 7.5, 1 mm EDTA, 1 mm DTT) and purified by ion exchange chromatography using a flow rate of 1 ml/min with a 1-ml HiTrap Q HP column (GE Healthcare) and eluting using a 20-ml gradient of 0–1 m NaCl in TED buffer. Fractions containing VirD2 were loaded onto a 16/60 Superdex 200-pg column, run at a flow rate of 0.5 ml/min, and eluted in 50 mm Tris, 200 mm NaCl, pH 7.5. The column was calibrated with known standards under equivalent conditions to produce a calibration curve and, therefore, estimates of molecular weight for the fractionated peaks (Bio-Rad). His_6_-tagged VirC1 protein was similarly expressed in BL21 pLysS and cells lysed in H buffer (50 mm Hepes, pH 7.5, 300 mm NaCl). After centrifugation (2 × 30 min at 4 °C), soluble proteins were incubated with 1 ml of Talon resin (Clontech). The column was washed with 50 ml of H buffer, and bound proteins were eluted in 4 ml of 50 mm NaOAc, pH 5, 300 mm NaCl. The eluate was centrifuged for 10 min to remove precipitated proteins, filtered, diluted with 45 ml of TED buffer, and loaded onto a 1-ml HiTrap heparin column run at a flow rate of 1 ml/min. Proteins were eluted with a gradient of 0–1 m NaCl in TED. Protein concentrations were determined using the BCA assay (Pierce).

##### DNA Binding Assay

Plasmid DNA was prepared from overnight cultures of *E. coli* XL1-Blue using a plasmid extraction kit (Qiagen). In standard 20-μl reactions, protein and 100 ng of DNA were mixed in binding buffer (20 mm Tris, pH 7.5, 5 mm MgCl_2_, 100 mm NaCl) and incubated at 37 °C for 30 min. To protease-treat products, 1 μl of 0.1% SDS and 1 μl of 20 mg ml^−1^ proteinase K (Sigma) were added, and incubation continued for an additional 30 min. Samples were subsequently mixed with loading dye (50% glycerol, 0.1% bromphenol blue) and immediately loaded onto a 0.8% agarose gel containing ethidium bromide.

##### Oligonucleotide Cleavage Assay

This method was as described previously (23) with modifications for use with digoxigenin (DIG)-labeled oligonucleotides (Sigma). Briefly, the labeled oligonucleotide (0.1 pmol) and protein were incubated in a 10-μl reaction containing buffer (20 mm Tris, pH 7.5, 5 mm MgCl_2_, 100 mm NaCl) for 2 h at 37 °C. Unlabeled competitor (100×) oligonucleotides C and N (10 pmol) were added where indicated. Samples were protease-treated by adding either 1 μl of 0.1% SDS plus 1 μl of proteinase K (20 mg ml^−1^) or 1 μl 0.1 m CaCl_2_ plus 1 μl 1× trypsin-EDTA solution (Sigma) and incubating for a further 30 min. Immediately after incubation, 10 μl of 2× sample buffer (12% Ficoll 400, 7 m urea, 0.1% (w/v) bromphenol blue, 0.1% (w/v) xylene cyanol in TBE) was added, and samples were denatured by heating to 70 °C for 3 min. Samples were resolved on denaturing 20% polyacrylamide, TBE 7 m urea gels run at 200 V for 100 min. DNA was then transferred to Hybond N^+^ and cross-linked by exposure to UV light. Labeled DNA was subsequently visualized using a DIG luminescent detection kit (Roche Applied Science).

##### Pull-down Assay

Fusion proteins were separately expressed in 250-ml cultures of *E. coli* for each pull-down experiment, harvested, and lysed by sonication in buffer A (50 mm Tris, 200 mm NaCl, 1 mm EDTA, 1 mm DTT, pH 7.5). Soluble protein lysates containing MBP or MBP fusions were clarified by centrifugation and then incubated with 0.5 ml of amylose resin in buffer A in a 2-ml Eppendorf tube for 90 min at 4 °C with mixing. The resin containing immobilized protein was subsequently washed 10 times with 1 ml of buffer A and then mixed with the soluble lysate from *E. coli* expressing His-VirC1. Resin was then applied to a mini-SpinX 0.22-μm cellulose acetate column (Costar) and washed 10 times by centrifugation with 0.5 ml of buffer A. The final wash flow-through was saved to confirm that no further unbound protein remained in the wash buffer. Immobilized proteins were finally eluted by the addition of 35 μl of buffer A containing maltose, and then 10 μl was resolved by 12.5% SDS-PAGE (Invitrogen) prior to Western immunoblot. Blots were probed with anti-His_6_ antibody (Novagen) and alkaline phosphatase-conjugated secondary antibody (Sigma) prior to signal detection using 5-bromo-4-chloro-3-indolyl phosphate/nitro blue tetrazolium liquid substrate (Sigma).

##### Yeast Two-hybrid Assay

The high efficiency lithium acetate transformation procedure (37) was used to co-transform relevant pGBT9 and pGAD424 constructs (20 μl each) into *S. cerevisiae* strain PJ69–4A. PJ69-4A contains three separate reporter genes (*HIS3*, *ADE2*, and *LacZ*), each under the independent control of three different *GAL4* promoters (*GAL1*, *GAL2*, and *GAL7*) that provide a high level of sensitivity with respect to detecting weak interaction coupled with a low background of false positives (38). Co-transformants were initially selected by plating on yeast minimal medium supplemented with 2% glucose (w/v) plus Met (20 μg ml^−1^), uracil (20 μg ml^−1^), His (20 μg ml^−1^), and Ade (20 μg ml^−1^) (MUHA plates) and then subsequently replica-plated onto yeast minimal medium minus His plus X-gal (MUAX plates) to select for activation of *HIS3*/*lacZ* reporters or onto yeast minimal medium minus His and Ade (MU plates) to select for activation of the *HIS3*/*ADE2* reporters. Quantitative assessment of β-galactosidase activity in PJ69-4A cell extracts as a secondary measure of *lacZ* reporter activity was made using *o*-nitrophenyl-β-d-galactopyranoside as substrate (39).

## RESULTS

### 

#### 

##### Sequence Analysis of Tfs4 VirD2

**FIGURE 1.**
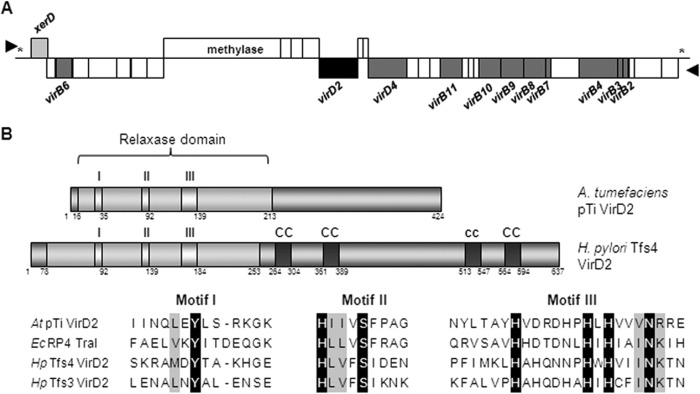
**Genetic context of *H. pylori tfs4 virD2* and conservation of relaxase motifs in the N terminus of the encoded protein.** *A*, schematic representation of the PZ *tfs4* gene cluster of *H. pylori* strain P12. The cluster encodes a putative VirD2-like protein (*black box*), VirD4 coupling protein, and a complement of VirB T4SS assembly proteins (*dark gray box*). The encoded XerD protein (*light gray box*) is a functional tyrosine-like recombinase that excises the chromosomal *tfs4* cluster at conserved 5′-AAAGAATG-3′ flanking motifs (indicated by *asterisks*). The direction of gene transcription is indicated by *arrowheads. B*, signature relaxase Motifs I–III are shown to be conserved within the N-terminal relaxase domain in both *H. pylori* strain P12 *tfs3-* and *tfs4*-encoded VirD2-like proteins (accession number CP0011217) in an alignment with MOB_P_ family relaxases represented by *A. tumefacians* pTiA6 VirD2 (accession number AF242881.1) and *E. coli* RP4 TraI (accession number X54459.1). The conserved catalytic tyrosine residue in Motif I is shown in *black highlight*. C-terminal Tfs4 VirD2 is not homologous to known sequences but comprises several regions with coiled-coil potential. Tfs3 VirD2 (amino acids 1–677) has a similar disposition of motifs as Tfs4 VirD2. Shown are confidently predicted coiled coil (COILS and Paircoil2) (*CC*) and coiled coil predicted by COILS only (*cc*). This figure was adapted from Ref. 41.

Two VirD2-like proteins can be identified within the genomes of some sequenced *H. pylori* strains based on sequence similarity in the N-terminal region of the proteins to the conserved VirD2 relaxase domain, COG3843 in the conserved domain database (*E* value = 1.19e−65). In strain P12, representative proteins are encoded by genes HPP12_1353 and HPP12_0451, the latter being located proximal to a complement of T4SS-encoding *vir* structural genes within the *tfs4* cluster (Fig. 1*A*). Both proteins contain conserved N-terminal relaxase motifs (I–III) (40, 41) (Fig. 1*B*) with sequence characteristics most closely resembling the MOB_P_ relaxase family, which includes the well studied relaxase TraI encoded on the *E. coli* plasmid RP4 and *Agrobacterium tumefacians* pTi VirD2 (34). However, overall, the proteins share minimal sequence identity; *A. tumefacians* pTi VirD2 has 9.8% identity and 20% similarity to Tfs4 VirD2 and 11.8% identity and 21% similarity to Tfs3 VirD2, whereas Tfs3 and Tfs4 VirD2 proteins share 20.5% identity and 33.2% similarity, although there is broadly comparable secondary structure in the N-terminal relaxase portion of the proteins (data not shown). The C-terminal region of Tfs4 VirD2 is not identified by similarity to known domains or sequences by PSI-BLAST search, although several regions with coiled-coil potential, absent in the C-terminal sequence of *A. tumefaciens* pTi VirD2 are predicted with confidence (COILS > 90% and Paircoil2 < 0.3; Fig. 1*B*).

##### Phylogenetic Analysis and Identification of a Putative tfs4 oriT Region

**FIGURE 2.**
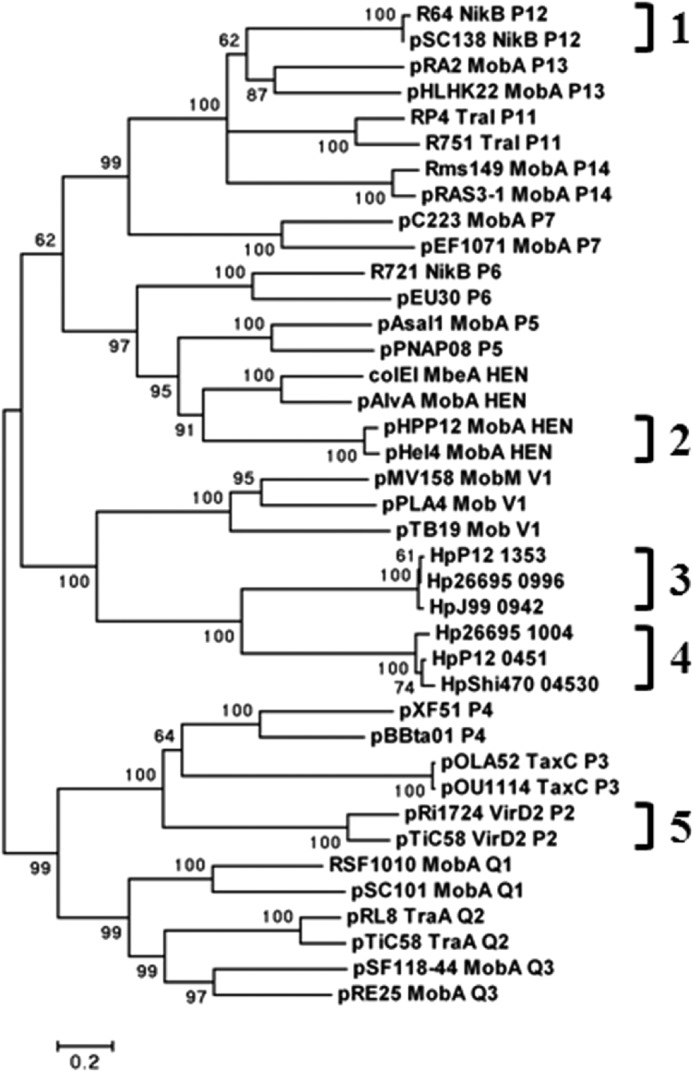
**Phylogenetic tree of relaxase families containing one active Tyr in the Motif I catalytic center.** Representative relaxase sequences were selected from the MOB families defined previously (34). The MOB_P_ family of relaxases incorporates the subclades P1–P7, P11–P14, Q1–Q3, and HEN, as indicated. The diverse MOB_V_ clade, of which the most populous MOB_V1_ subclade is shown, is ancestrally related to the MOB_P_ family. *H. pylori* Tfs3 (*3*) and Tfs4 (*4*) VirD2-like relaxases are shown not to be contained within established MOB clades. MOB_P_ subclades containing plasmid RP4 (*1*), endogenous *H. pylori* plasmids (*2*) and *A. tumfaciens* VirD2 relaxases (*5*) are indicated. The percentage of trees (2000 in total) supporting each node is shown.

To define the relationship between *tfs3/tfs4*-encoded VirD2 proteins with established MOB relaxase families, a phylogenetic analysis was performed using the N-terminal sequence (1–300 amino acid residues) of 33 relaxases representative of the main MOB families that share the characteristic of a single Motif I active site tyrosine residue (34). The resulting phylogeny (Fig. 2) indicates that, although Tfs3 and Tfs4 VirD2 proteins have sequence-conserved relaxase motifs strongly reminiscent of the MOB_P_ family of relaxases, together with the MOB_V_ clade, they are more ancestrally remote and are not obviously classified within the established MOB clusters.

**FIGURE 3.**
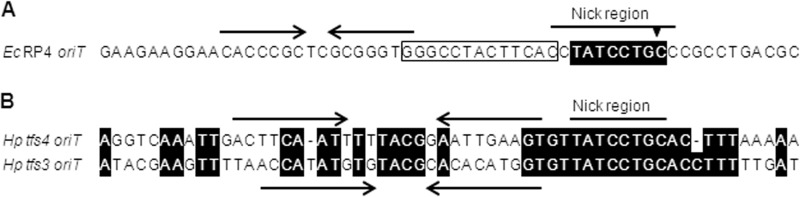
**Comparison of the *E. coli* RP4 plasmid *oriT* with putative PZ *oriT* nick regions.** *A*, sequence of the well characterized RP4 *oriT* region highlighting the 16-bp inverted repeat (*arrows*) immediately proximal to the nick region comprising the conserved MOB_P_ family core nick sequence 5′-ATCCTG-3′ (position of *nic* cleavage site indicated by a *triangle*). The binding site of the RP4 TraI relaxase is shown within a *box* adjacent to the core conserved nick sequence (*shaded*). *B*, sequence conservation of *tfs4* and *tfs3* intergenic *oriT* regions upstream of encoded VirD2-like proteins. Perfect (*tfs4*) and imperfect (*tfs3*, one mismatch) 9–10-bp distal and proximal arms (*arrows*) of a 25-bp inverted repeat characteristic of *oriT* regions are evident immediately 5′-proximal to the putative *tfs3* and 5′-ATCCTG-3′ *tfs4* nick region. The *tfs4* sequence shown is invariantly conserved between both P12 and AB21 strains. *Shading* highlights sequence identical to the *tfs4* region.

Relaxases of the same MOB family and motif signature often recognize and nick within the same cognate *oriT* sequence (34) comprising both a highly conserved nick region sequence and associated upstream inverted repeat, the latter being more variable in sequence and containing binding sites for both relaxase and auxiliary relaxosome proteins (42) (Fig. 3*A*). The MOB_P_ family *A. tumefaciens* VirD2 (MOB_P2_) and TraI of the conjugative plasmid RP4 (MOB_P11_) both require the core hexanucleotide sequence 5′-ATCCTG-3′ for cleavage activity *in vitro*, although a consensus nick sequence can be derived that extends to additional flanking bases, 5′-(C/T)ATCCTG(C/T)-3′ (29, 40, 43). Although Tfs4 VirD2 appears phylogenetically distinct from the MOB_P_ family, its relaxase motifs are highly conserved relative to the MOB_P2/P11_ subclades (Fig. 1). As such, we speculated that it might therefore have similar substrate sequence specificity and, as a self-transmissible genomic element (12), would necessarily also contain an *oriT* sequence for the initiation of transfer. We therefore searched for a MOB_P_ family consensus motif within the *tfs4* gene cluster of strain P12. Three such sequences were apparent, two within the coding sequence of *xerD* and *virB10* and the other in an intergenic region immediately upstream of the coding sequence of *virD2*. Further examination of the intergenic sequence identified a perfect 25-bp inverted repeat immediately proximal to the putative 5′-TATCCTGC-3′ nick motif, providing this region with features characteristic of an *oriT* sequence (Fig. 3). Notably, an equivalent *oriT*-like sequence comprising an identical MOB_P_ nick motif was also identified upstream of the *virD2* in the PZ *tfs3* cluster (Fig. 3). BLAST alignments determined that the 104-bp intergenic sequences incorporating the putative PZ *oriTs* (Fig. 3) are invariantly conserved in the majority of *H. pylori* strains for which sequence is presently known (*tfs4* sequence invariant in 20 strains (5e−20) and the *tfs3* sequence in 11 strains (4e−21)), further alluding to the functional significance of this region.

##### Expression and Purification of Tfs4 VirD2

**FIGURE 4.**
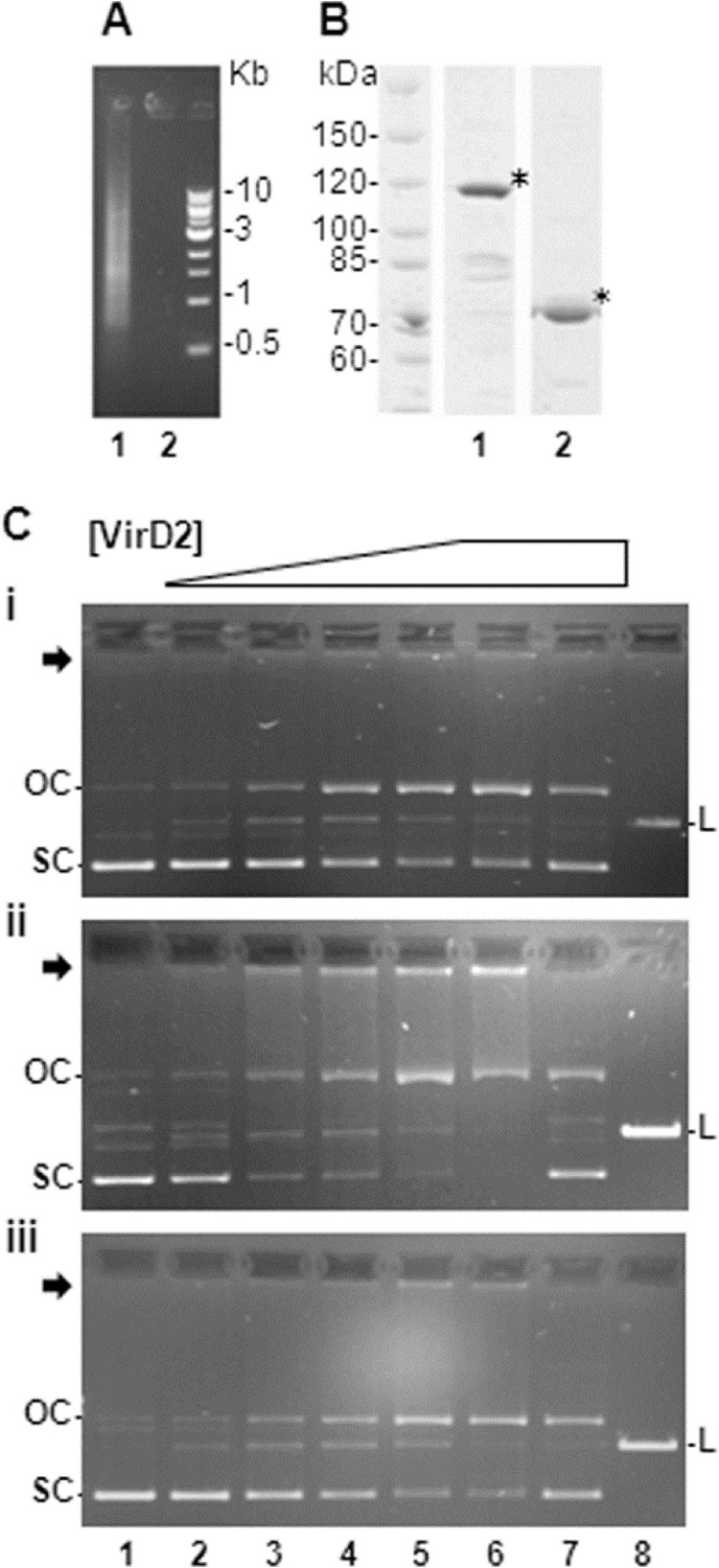
**Purification and DNA binding activity of Tfs4 VirD2.** *A*, DNA co-fractionating in the MBP-VirD2 size exclusion chromatography fraction (*lane 1*) is efficiently removed in a subsequent ion exchange step (*lane 2*). *B*, three-stage purification of MBP-VirD2. *, purified MBP-Tfs4 VirD2 (*lane 1*) and MBP-Tfs4 VirD2(N) (*lane 2*) resolved by 10% SDS-PAGE. *C*, MBP-VirD2 DNA binding activity. Incubation of MBP-VirD2 with 100 ng of pSB14 (containing cloned RP4 *oriT*) (*i*), pRD205 (pGEM-TEasy containing cloned *tfs4 virD2* and upstream intergenic sequence) (*ii*), or pRD200 (pGEM-TEasy containing cloned *tfs4 virD2* only) (*iii*) results in a decrease in supercoiled plasmid and concomitant increase in both open circle (nicked) forms and loading well-retarded nucleoprotein complexes (*black arrow*) as protein concentration increases. Effects are most pronounced with plasmid pRD205. Notably, linear plasmid is also absent from the pRD205 sample following proteinase K treatment. *Lanes 1–6*, 0, 0.05, 0.1, 0.15, 0.3, and 0.5 pmol of MBP-VirD2; *lane 7*, 0.5 pmol of MBP-VirD2 treated with proteinase K; *lane 8*, linear plasmid generated by restriction enzyme digest. All reactions were performed at 37 °C in the presence of MgCl_2_. *OC*, open circle (nicked); *SC*, supercoiled, *L*, linear.

Homologues of P12 *tfs4 virD2* genes were identified in a selection of clinical isolates from our strain collection by PCR typing, and then sequences for both full-length protein (amino acids 1–637) and the N-terminal relaxase domain (amino acids 1–257) were cloned and expressed in *E. coli* as N-terminal maltose-binding protein (MBP) fusion proteins to enhance solubility and stability; initial efforts to express equivalent VirD2 proteins with a minimal His tag resulted in low levels of expression of unstable and almost entirely insoluble protein. Expressed MBP fusion proteins were subjected to a three-stage purification protocol in which they were first purified by affinity chromatography, fractionated by size exclusion chromatography, and finally purified in an ion exchange separation step. The latter step was required to remove co-purifying DNA from the size exclusion MBP-VirD2 fractions (Fig. 4*A*). Of note, both full-length VirD2 and N-terminal domain MBP fusions (MBP-VirD2 and MBP-VirD2(N), respectively) were found to elute in the void volume during size exclusion chromatography, suggesting protein aggregation, possibly due to the presence of contaminating DNA or the formation of quaternary complexes much larger than the predicted ∼121-kDa (MBP-VirD2) or ∼76-kDa (MBP-VirD2(N)) purified monomeric MBP-VirD2 fusion proteins observed by SDS-PAGE (Fig. 4*B*).

##### Tfs4 VirD2 Strand-specific Relaxase Activity

Several relaxases, including TrwC of plasmid R388 and TraI of F plasmids (both MOB_F_ family), bind and nick at their cognate *oriT in vitro* in the absence of auxiliary factors, requiring only the presence of Mg^2+^ and supercoiled plasmid DNA (scDNA) for nicking activity (31, 44). To assess the activity of Tfs4 VirD2 in this context, we examined the general effects of incubating purified MBP-VirD2 with a selection of plasmid DNAs, each containing a different putative nick region using an electrophoretic mobility shift assay. Plasmids, prepared by conventional alkaline lysis, included the *H. pylori* shuttle vector pSB14 containing a cloned RP4 *oriT* (45) and two pGEMT-based vectors, one containing cloned *tfs4 virD2* plus the putative upstream intergenic *oriT* sequence (pRD205) and the other containing just *tfs4 virD2* (pRD200). In all cases, incubation of plasmid (100 ng) with increasing amounts of MBP-VirD2 (0.05–0.5 pmol) resulted in a concentration-dependent conversion of scDNA to both the open circle, nicked form and a non-migrating species retarded in the gel loading wells (Fig. 4*C*, *lanes 2–6*). Subsequent treatment with detergent and protease (Fig. 4*C*, *lane 7*) released plasmid from wells as both supercoiled and nicked species, confirming the non-migrating plasmid to be in the nucleoprotein complex. Effects were most prominently observed with pRD205 containing the putative *tfs4 oriT*, and notably, whereas a small and broadly constant amount of linear product was observed in all incubations regardless of MBP-VirD2 concentration, pRD205 was the only plasmid in which linear product was no longer evident following protease treatment and release from VirD2 binding (Fig. 4*C*, *ii*). This suggests that in complex with linear plasmid containing a particular *oriT*, an excess of VirD2 can mediate an end-joining reaction *in vitro*, resulting in resealing of the phosphodiester backbone. All effects required the presence of MgCl_2_ and were not observed when plasmid was incubated with MBP alone (data not shown). Collectively, these results demonstrate that, in limiting concentration, *tfs4* VirD2 can reversibly bind scDNA independently of other factors *in vitro* and catalyze a strand-specific nicking reaction that is dependent upon the presence of Mg^2+^. An excess of protein, however, results in seemingly irreversible formation of large nucleoprotein complexes or aggregates requiring protein denaturation for plasmid release.

##### Sequence-specific Cleavage of Oligonucleotides by Tfs4 VirD2

The sequence specificity for Tfs4 VirD2 binding and nicking activity could not be determined from the previous experiments because all plasmids contain the 5′-ATCCTG-3′ hexanucleotide in their backbone sequence in addition to the putative nick motifs within the cloned *oriT* regions. Therefore, to more clearly demonstrate nicking activity of Tfs4 VirD2 and to determine its target sequence requirements, two 30-base substrate oligonucleotides were designed for use in single-stranded DNA cleavage assays. The first was based on the putative *tfs4 oriT* nick sequence (“Tfs4”) invariantly conserved in unrelated *H. pylori* strains AB21 and P12, and a second was based on the RP4 *oriT* sequence (“RP4”) identical to the cloned fragment within pSB14 (45). Because relaxase activity is strictly a component of the N-terminal relaxase domain (46–48), we employed an MBP fusion to N-terminal VirD2, MBP-VirD2(N), in these assays to confine observations to this region of the protein. Subsequent cleavage products resulting from incubation of MBP-VirD2(N) with DIG-labeled oligonucleotides were separated in denaturing polyacrylamide gels and analyzed by Southern blotting.

**FIGURE 5.**
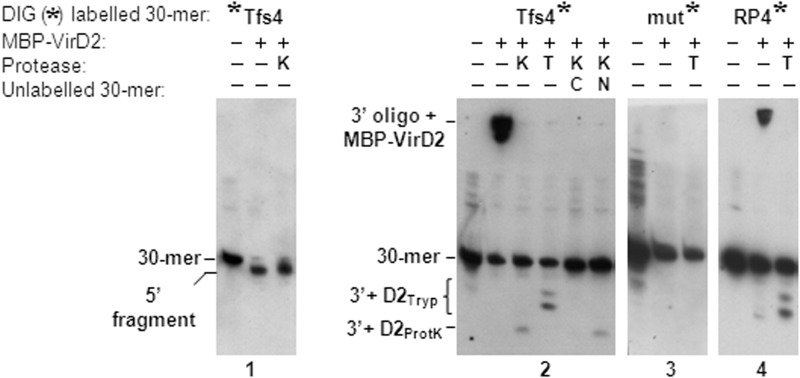
**Site-specific cleavage of oligonucleotides by Tfs4 VirD2.** The indicated 5′ (Tfs4) or 3′ (Tfs4, Tfs4 mutated (mut), and RP4) DIG-labeled 30-mer oligonucleotides were incubated with 5 pmol of Tfs4 MBP-VirD2(N) and then subsequently in the presence or absence of either Proteinase K (*K*) or trypsin (*T*). The resulting oligonucleotide products and nucleoprotein-peptide complexes were resolved in denaturing 20% polyacrylamide gels and analyzed by Southern blotting. MBP-VirD2(N) cleaves the 3′-end of the 5′-DIG-labeled Tfs4 oligonucleotide (putative *oriT* ATCCTG-containing sequence upstream of *virD2* in the *tfs4* cluster) (*blot 1*). The equivalent 3′-DIG-labeled oligonucleotide is retained in the gel well in the presence of MBP-VirD2(N) (*blot 2*). Following protease treatment, cleaved ATCCTG-containing oligonucleotides demonstrate retarded gel migration due to the attachment of proteolyzed VirD2 peptides (D2_Tryp_ and D2_ProtK_, *blots 2* and *4*). Cleavage and VirD2 peptide attachment to 3′-DIG-labeled oligonucleotides can be effectively abrogated by the addition of a 100-fold excess of competing unlabeled Tfs4 oligonucleotide (*C*) but not by the addition of non-competing random sequence oligonucleotide lacking the ATCCTG sequence (*N*) (*blot 2*). Cleavage is similarly not observed following incubation of MBP-VirD2(N) with a Tfs4 3′-DIG-labeled oligonucleotide in which the ATCCTG sequence is entirely mutated (*mut*; *blot 3*). All reactions required the presence of MgCl_2_. Full oligonucleotide sequences are listed in Table 1.

Incubation of MBP-VirD2(N) with 5′ DIG-labeled Tfs4 *oriT* oligonucleotide resulted in cleavage of the 30-mer oligonucleotide to a marginally smaller product, indicating loss of a small (<10 nucleotides) 3′-unlabeled fragment. That mobility of the large 5′-labeled fragment was not retarded in the gel indicates that VirD2 does not bind to the 3′-cleaved end and further, given the large size of this product, that cleavage occurs either within or immediately 3′ of the 5′-ATCCTG-3′ sequence (Fig. 5, *blot 1*), located 9 nucleotides from the 3′-end of the Tfs4 oligonucleotide (Table 2).

**TABLE 2:** **Oligonucleotides**

Name	Oligonucleotide sequence*^a^*
virD2F1	AAGGATCCATGGCGTTAGAAAAAAGTTATAGTAA
virD2R1	AAGGATCCTTAAAAATCCATTTCATAGTTATTGTTAG
virD2R2	AAGGATCCTTAAAAACTCTTATTGTTTCTAGCCTGTTTTTC
DIG -tfs4	DIG-CGGAATTGAAGTGTTATCCTGCACTTTAAA
tfs4-DIG	CGGAATTGAAGTGTTATCCTGCACTTTAAA-DIG
mut tfs4-DIG	CGGAATTGAAGTGTTGCATGTCACTTTAAA-DIG
RP4 oriT-DIG	GGGCCTACTTCACCTATCCTGCCCGGCTGA-DIG
Competitor	CGGAATTGAAGTGTTATCCTGCACTTTAAA
Non-competitor	AAGGATCCAAAACTCTTATTGTTTCTAGCCTGTTTTTC
virD4F	CGTTTTATGCAAGTCTTTACAAG
virC1F	CATGCCATGGGCCATCATCATCATCATCACGGTATAATCACCATAGCTAATGAAAAAGGA
virC1R	GCGGGATCCTCATTTTTTGATCCTTAAATACCCG
Y2HvirD2F	CGATGGATCCTGGCGTTAGAAAAAAGTTATAGTAAAG
Y2HvirD2R	AAGGATCCTTAAAAATCCATTTCATAGTTATTGTTAG
Y2HvirC1F	CCGGAATTCATAATCACCATAGCTAATGAAAAAGGA
Y2HvirC1R	GCGGGATCCTCATTTTTTGATCCTTAAATACCCG
Y2H4520F	CGTAGAATTCGAATTTAAAAACACTAAAAAAGACAGG
Y2H4520R	TTCGGGGATCCTCACAACTTGCTCGCCTTATC
Y2H4525F	CGTAGAATTCAAGCAAATTTTAAACGACCACTT
Y2H4525R	TTCGGGGATCCTTACTTAGCGTATTTTTTAACCAATTC

*^a^* The sequence corresponding to the core 6-bp nick region is underlined where appropriate. DIG, digoxigenin immunolabel.

Conversely, incubation with the identical 3′ DIG-labeled 30-mer oligonucleotide resulted in a non-migrating product observed in the gel well, corresponding to the 3′-cleavage fragment attached at its 5′-end to MBP-VirD2(N) protein. Subsequent treatment of the retarded nucleoprotein complex with proteases liberated the small (∼9-mer) 3′-oligonucleotide cleavage product, the gel mobility of which differed according to the size of the trypsin or proteinase K-proteolyzed peptide fragment of VirD2 remaining attached (Fig. 5, *blot 2*, *lanes 3* and *4*). In competition experiments, both VirD2 binding to and nicking of the labeled substrate could be inhibited by adding a 100-fold excess of unlabeled competing Tfs4 oligonucleotide but was not affected by the presence of excess unlabeled random sequence oligonucleotide, confirming nick sequence specificity of the Tfs4 VirD2 active site to sequence within the Tfs4 oligonucleotide (Fig. 5, *blot 2*, *lanes 5* and *6*). Binding and nicking activity was subsequently determined to be specifically dependent upon the 5′-ATCCTG-3′ hexanucleotide sequence by lack of discernable VirD2 activity toward a DIG-labeled Tfs4 oligonucleotide with a 6-position base-substituted 5′-ATCCTG-3′ sequence (Fig. 5, mut/blot 3a). Finally, an oligonucleotide containing the RP4 *oriT* nick region (RP4), comprising the MOB_P_ family consensus motif in an entirely different flanking sequence context, was also nicked by Tfs4 VirD2. RP4 and Tfs4 oligonucleotide cleavage products appeared identical, indicating cleavage at the same position within the RP4 nick sequence, 5′-TATCCTGC-3′, common to both Tfs4 and RP4 oligonucleotides. Tfs4 VirD2 is therefore indicated to have sequence-specific nicking activity, which, similar to a subset of non-MOB_P_ family relaxases, is independent of auxiliary factors *in vitro*, and a nick sequence specificity that conforms to that of the MOB_P_ family consensus motif, consistent with the character of its signature relaxase motifs.

##### Prevalence of the Tfs4 VirD2 Nick Sequence

The collective results of the cleavage assays determine that Tfs4 VirD2 specifically recognizes the conserved hexanucleotide nick motif 5′-ATCCTG-3′ *in vitro*. Because certain ICEs have been demonstrated to mobilize chromosomal DNA, plasmids, and other GIs that lack machinery for self-mobilization (27, 49), we considered whether there might be additional cognate nick sites outside of the *tfs4* PZ cluster that might be subject to Tfs4 VirD2 activity. To investigate this, the consensus sequence (C/T)ATCCTG(C/T), incorporating the sequence context of both the putative *tfs4/tfs3 oriT* nick sequence and known nick regions of MOB_P_ family relaxases, was used as a search thread to interrogate the genome sequence of strain P12. Accounting for both strands, 69 sites in total were identified, 24 of these comprising the conserved 8-bp 5-TATCCTGC-3′ sequence of the putative *tfs4 oriT* nick motif (supplemental Table 1).

Next, to define these regions as candidate *oriT* regions specifying for *in vivo* relaxase activity, the first 50-bp sequence upstream of each putative nick motif was assessed for the presence of inverted repeats using the EMBOSS Palindrome program set to detect palindromes of 8 bp or more with one permissible mismatch. Using this criterion, which reflects the sequence and motif disposition of RP4, *A. tumefaciens* pTi, and the putative *tfs4 oriT* regions, seven sequence regions were identified. However, of these, only *tfs4* and *tfs3 oriT* regions were intergenic, suggesting that PZ relaxase activity *in vivo* may be restricted to these specific chromosomal regions, at least in strain P12. Interestingly, the 8-bp *tfs4* nick motif was also evident in the endogenous pHPP12 plasmid and also conserved in several other *H. pylori* plasmids (supplemental Table 1). However, it was not present in all *H. pylori* plasmids and was contained within coding sequence, and the inverted repeat was present in the immediate 3′- rather than 5′-proximal flanking sequence.

##### Identification of a Putative VirC1 Protein and Interaction with VirD2

Elaboration of relaxase function *in vivo* occurs in the context of the relaxosome complex of auxiliary proteins, which both assist relaxase-mediated cleavage at the cognate *oriT* and recruitment of relaxase-bound transfer intermediates to the membrane-localized secretion machinery (21, 28, 30–32). In *Agrobacterium*, the relaxosome comprises VirD1, VirD2, VirC1, and VirC2 (32). Of these, the ParA/MinD-like ATPase protein, VirC1, mediates relaxosome formation at the *oriT*-like border sequences and coordinates transfer of nucleoprotein complexes to the secretion channel (32).

**FIGURE 6.**
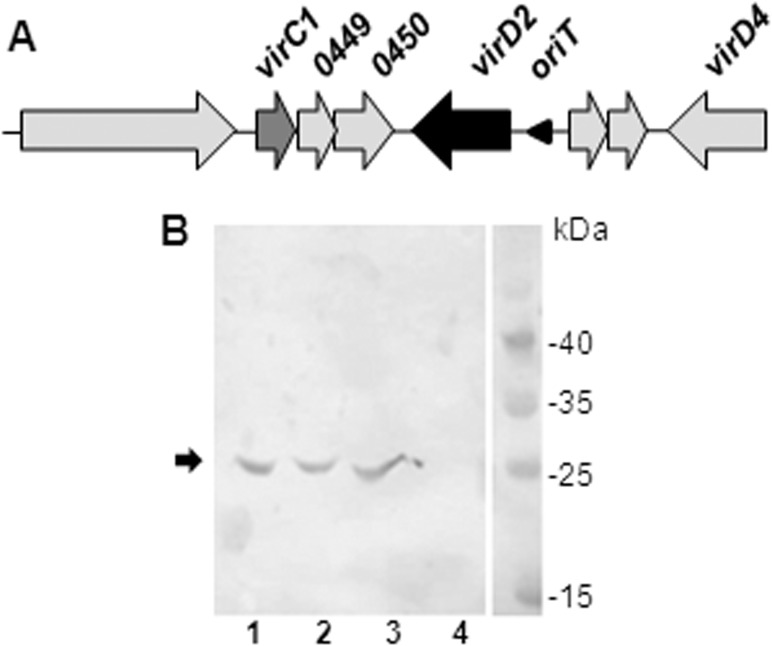
**Tfs4 VirD2 interaction with a putative VirC1 homologue.** *A*, gene arrangement in the vicinity of *virD2* within the *H. pylori* P12 *tfs4* gene cluster (12). Genes encoding VirD2 and VirC1 are highlighted by *black* and *dark gray shading*, respectively, and the intergenic position of the putative *tfs4 oriT* sequence is indicated by a *triangle. B*, pull-down assay demonstrating Tfs4 VirD2-VirC1 protein-protein interaction. Whole-cell lysates containing MBP-VirD2, MBP-VirD2(N), or MBP alone were immobilized on amylose resin, washed thoroughly, and then mixed with a soluble cell lysate containing His-tagged Tfs4 VirC1. Following a further 10× column volume wash step, immobilized proteins were eluted, and samples were analyzed by 12.5% SDS-PAGE and Western immunoblot using anti-His_6_ antibodies (Novagen). His-tagged VirC1 (indicated by an *arrow*) is shown to be present in the soluble cell lysate prior to incubation with MBP proteins (*lane 1*) and is specifically co-eluted with amylose resin-immobilized MBP-VirD2 (*lane 2*) and MBP-VirD2(N) (*lane 3*) but not MBP alone (*lane 4*).

In *H. pylori tfs4*, a *virC1* homologue (gene *0448*) can be identified as the first of three contiguous genes convergent with *virD2* (Fig. 6*A*). The encoded protein shares 22.8% identity and 39.8% similarity with *A. tumefacians* VirC1 and has the conserved domain structure and ATPase motifs characteristic of the ParA, VirC1, and RP4 TraL family (50). The two other genes comprising the putative *virC1* operon (homologues of genes *0449* and *0450* in the P12 genome) are of unknown function, appearing unique to *H. pylori tfs3*/*tfs4* clusters.

Because VirC1 proteins are demonstrated to interact with VirD2 and other relaxosome components, we first employed the yeast two-hybrid assay to investigate the possibility of equivalent interactions between the Tfs4 VirD2 and VirC1-like proteins. Because genes encoded within the same operon often function in the same biological context, we also included *0449* and *0450* in our yeast two-hybrid screens as additional candidate components of a Tfs4 relaxosome. The four sequences were cloned into yeast two-hybrid vectors pGAD424 (GAL4 activation domain fusion/prey vector) and pGBT9 (GAL4 binding domain fusion/bait), and all heterologous bait/prey pairwise combinations were co-transformed into *S. cerevisiae* strain PJ69-4A (38). Positive interactions were indicated by activation of reporter combinations (*HIS3*, *ADE2*, and *lacZ*), enabling direct assessment of the yeast two-hybrid phenotype by the color of transformant colonies growing on minimal selective medium, and subsequently also by a β-galactosidase assay. None of the fusions were found to self-activate yeast reporters in control transformations. Pairwise interaction screens indicated reciprocal VirD2-VirD2 and VirD2-VirC1 interactions both by stringent growth selection and a β-galactosidase assay (Table 3). No other interactions were strongly predicted, although non-reciprocal activation of two reporters suggested possible weak or transient interaction between VirD2–0449 and VirD2–0450 (Table 3).

**TABLE 3:** **Results of yeast two-hybrid analysis**

	VirD2*^a^*				VirC1*^a^*			0449*^a^*			0450*^a^*		
	VirD2*^b^*	VirC1*^b^*	0449*^b^*	0450*^b^*	VirD2*^b^*	0449*^b^*	0450*^b^*	VirD2*^b^*	VirC1*^b^*	0450*^b^*	VirD*^b^*	VirC1*^b^*	0449*^b^*
−His/−Ade*^c^*	+	−	−	−	+	−	−	−	−	−	−	−	−
−His*^c^*	+	+	+	+	+	+	+	+	+	+	+	+	+
*lacZ**^d^*	+	+	−	−	+	−	−	+	−	−	+	−	−

*^a^* Prey fusions were constructed in the pGAD424 vector.

*^b^* Bait fusions were constructed in the pGBT9 vector.

*^c^* YMM plates were supplemented with Met and uracil and lacked either His or both His and Ade, as indicated.

*^d^ lacZ* reporter activity was assessed both by blue colony color on 5-bromo-4-chloro-3-indolyl-β-d-galactopyranoside plates and subsequently by a β-galactosidase assay.

To provide biochemical evidence in support of the VirD2-VirC1 interaction, we analyzed binding of VirC1, expressed as a soluble His_6_-tagged protein in *E. coli*, to either MBP or MBP-VirD2 and MBP-VirD2(N) fusions immobilized on amylose resin. Subsequent Western immunoblot analysis of eluted proteins using anti-His_6_ tag antibodies showed co-elution of His-VirC1 with both MBP-VirD2 fusions but not with MBP alone (Fig. 6), providing secondary evidence in support of a specific Tfs4 VirD2-VirC1 interaction. Notably, the N-terminal relaxase domain of VirD2 appears sufficient for the interaction with VirC1.

## DISCUSSION

Relaxase proteins are key essential components in the processing and mobilization of bacterial DNA via conjugative mechanisms. Commonly, they mediate the transfer of endogenous plasmids between strains but are also integral to the dissemination of self-transmissible mobile genetic elements, such as conjugative transposons and ICEs (51). The *tfs4* PZ gene cluster is described as a self-transmissible genomic island/conjugative transposon (11, 12), although its function and clinical relevance remains unclear, particularly because it appears to be inactive in many strain backgrounds due to either fragmentation or inactivating mutation. However, interstrain transfer of large fragments of the *tfs4* gene cluster has recently been demonstrated (12), suggesting a mechanism for rapid reconstitution of inactive T4SSs and alluding to a significant, but perhaps sporadic, benefit for maintenance of Tfs4 T4SS capability within the *H. pylori* population. Because relaxase activity would probably be critical for this process, we sought to examine the functional activity and biochemical properties of a VirD2-like relaxase encoded within the *tfs4* cluster.

As noted for a homologous protein (HP1004) in an early *in silico* analysis of reference strain 26695 (41), the protein we define here as Tfs4 VirD2 comprises a well defined N-terminal relaxase domain with relaxase sequence motifs (I–III; Fig. 1) similar to the well characterized conjugative RP4 TraI and *A. tumefaciens* VirD2 proteins of the MOB_P_ superfamily. Surprisingly, however, despite an evident ancestral relationship to these proteins, Tfs4 VirD2, together with Tfs3 VirD2, appear phylogenetically distinct and are not clearly assigned to any of the established MOB families. In this respect, the PZ VirD2 proteins are quite atypical because distinct clades and even subclades within the same MOB relaxase family invariably display different patterns of signature sequence conservation within component relaxase motifs (34).

Nevertheless, consistent with the sequence specificity of many MOB_P_ family relaxases for the consensus 5′-(C/T)ATCCTG(C/T)-3′ *oriT* nick sequence, Tfs4 VirD2 also demonstrates classical metal ion (Mg^2+^)-dependent relaxase activity at this core motif; following cleavage, the protein becomes tightly attached to the 5′ terminus of the nicked fragment and remains attached as a peptide fragment following proteolytic digest. Conventionally, this interaction is mediated by a phosphotyrosyl linkage between the relaxase Motif I active site tyrosine residue and the 5′ DNA terminus at the *nic* cleavage site (20–23) and appears consistent with our observations for Tfs4 VirD2. Indeed, the ability of purified Tfs4 VirD2 to specifically nick DNA *in vitro* in the absence of any other factors is a clear demonstration that it contains the active site required for phosphodiester bond cleavage at the *nic* site.

Cleavage of oligonucleotides containing an appropriate nick region in the absence of other relaxosome proteins is a commonly reported *in vitro* activity of purified relaxase proteins. However, nicking of duplex plasmid DNA containing equivalent nick sequences invariably requires the additional presence of one or several auxiliary relaxosome proteins and protease treatment to observe conversion of supercoiled plasmid to nicked forms (29). Tfs4 VirD2 differs somewhat in these respects; although all Tfs4 VirD2 nicking activity requires Mg^2+^ and supercoiled plasmid, conforming to requirements of other relaxases (20–23, 29, 44), nicking is observed entirely independently of other proteins and, more unusually, protein denaturant. This latter observation indicates that at low concentrations, the association of Tfs4 VirD2 with duplex DNA is more transient than observed for other relaxases, allowing for release of protein-free nicked intermediate following single strand cleavage.

Characteristically, relaxases that function to mobilize plasmids exhibit a long half-life in DNA complex (52). Although the shorter half-life of the Tfs4 VirD2-DNA interaction seen here is clearly a component of protein concentration, the fact that it is observed at limiting concentrations of VirD2 suggests it to be functionally significant. At higher concentrations, plasmid is seen to be increasingly bound in more stable, if not irreversible, nucleoprotein complex (Fig. 4*C*, *ii*), which, as suggested by size exclusion chromatography, may be explained by a tendency toward VirD2 aggregation or multimerization at higher protein concentrations *in vitro*. Although nicked plasmid is clearly evident at low VirD2 concentrations in the absence of denaturants, that protease treatment of nucleoprotein complexes recovers both nicked and more topologically constrained (supercoiled) forms suggests that when in complex with Tfs4 VirD2, plasmid is in equilibrium between nicked and ligated states, as proposed previously (29). Resealing of the phosphodiester backbone is a complementary activity of relaxase function necessary for termination of DNA strand transfer and, in the case of relaxases with a single active site tyrosine, usually requires relaxase dimerization (53). Consistently, the yeast two-hybrid analyses indicate that Tfs4 VirD2 may also dimerize, although a propensity for homomultimerization of purified protein *in vitro* is also observed. Interestingly, we also observed linearization of plasmid upon incubation with even the lowest concentration of Tfs4 VirD2. Cleavage of both DNA strands may reflect nonspecific activity, as similarly observed for the BmpH Mob protein of the Tn*5520* mobilizable transposon (54) and for the Orf20 relaxase of the conjugative transposon Tn*916* when incubated with DNA in the absence of an auxiliary specificity protein (24). A similar requirement may contribute to the residual *in vitro* activity of Tfs4 VirD2 seen here. More remarkably, whereas the linear species appeared to diminish at higher VirD2 concentrations, it was entirely absent from protease-treated VirD2-pRD205 complexes (Fig. 4*C*, *ii*), suggesting that when in nucleoprotein complex, *in vitro* at least, VirD2 also has a capacity for rejoining of both single and double DNA strands. Whether these observations represent novel catalytic activity of Tfs4 VirD2 or, more simply, artifactual *in vitro* activity resulting from a saturating concentration of fusion protein in high molecular weight nucleoprotein complex remains to be determined. With respect to the latter possibility, nonspecific cleavage of duplex DNA *in vitro* appears to be most notably associated with transposon mobilization proteins (24, 54), and it may therefore be the case that the observed atypical Tfs4 VirD2 activities are reflective of subtle functional differences, prominent *in vitro*, of a non-plasmid class of relaxase.

Although Tfs4 VirD2 bound and nicked all plasmids in this study, it appeared to have the most pronounced effect on supercoiled pRD205, comprising the putative *tfs4 oriT,* within the comparable concentration range used. Because the 5′-ATCCTG-3′ motif was present in all templates, we consider that the enhanced activity toward pRD205 was specifically a component of the broader sequence context of the cloned *tfs4 oriT.* Although *in vitro*, the nick-region proximal inverted repeat probably offers optimal tight positional binding for Tfs4 VirD2 nicking, a previous observation that an N-terminal fragment of Tfs4 VirD2 (termed Rlx2) expressed in *trans* could not be demonstrated to nick the cloned RP4 *oriT* in pSB14 by a primer extension assay (45) suggests involvement of other factors for nick region targeting *in vivo*. In this respect, we also demonstrated direct interaction between Tfs4 VirD2 and a putative *tfs4*-encoded VirC1 homologue. In the T-DNA transfer system of *A. tumefaciens*, the VirC1 ATPase protein nucleates relaxosome formation at *oriT* by directly interacting with VirD2 and other relaxosomal proteins and has a further role in recruitment of transfer intermediates to the T4SS channel-associated coupling protein (32). By analogy, we speculate that the Tfs4 VirC1 homologue may fulfill similar functions at the *tfs4 oriT*, possibly in association with additional yet-to-be-confirmed relaxosome components.

As potential specificity determinants for Tfs4 VirD2 activity, both auxiliary proteins and cognate *oriT* would conventionally function to selectively target relaxase activity to one specific nick sequence of the many identical or similar sequences encoded within the genome. Consistently, we found a modest distribution of candidate *oriT*s within the *H. pylori* genome, suggesting that activity of the PZ-encoded relaxases is specifically targeted to the *tfs3* and *tfs4* clusters and that they most likely function in mobilization of these regions. Because both PZ clusters and endogenous plasmids each encode an associated relaxase and sequence diverse *oriT* sequences, albeit with the same conserved nick region, we speculate that reciprocal relaxase activity at even these similar *oriT* sequences may not be permissible *in vivo*. In this respect, mobilization of endogenous plasmids by PZ T4SSs has not been demonstrated (8, 12).

Transfer of segments of the *tfs4* cluster has been shown to be dependent upon the function of the *tfs4*-encoded XerD tyrosine-like recombinase for chromosomal excision (12). Our data indicate that the VirD2-like relaxase will also be integral to this process and, via activity at *oriT*, may initiate transfer of PZ genes in a manner similar to ICE mobilization. ICEs typically also encode an integrase, a relaxase, and a T4SS required for ICE transfer via the T4SS generated mating pore (51). Following integrase-mediated ICE excision, the resulting extrachromosomal single-stranded circular ICE intermediate is nicked by the relaxase at an intergenic *cis*-acting *oriT* locus. The relaxase attached to the 5′-end of the single-stranded DNA is subsequently recruited to the coupling protein component of the mating pore and then transferred in a T4SS-dependent manner to a recipient cell (51). By close analogy, *tfs3* and *tfs4* circular intermediates generated by activity of the associated PZ XerD recombinase (12) can be predicted to follow a similar pathway mediated by the respective PZ relaxase acting at its cognate *oriT* within the excised PZ clusters. That PZ *tfs3* and *tfs4* clusters additionally encode a complement of Vir-homologous T4SS structural proteins, including a putative VirD4 coupling protein, indicates that these regions similarly comprise all of the elements required for self-transmissibility.

## Footnotes

2 The abbreviations used are:

PZ: plasticity zone
*oriT*: origin of transfer
ICE: integrating conjugative element
T4SS: type IV secretion system
MBP: maltose-binding protein
DIG: digoxigenin
scDNA: supercoiled DNA.
